# Explanation Beyond Individual Features: Instance-wise Feature Grouping for EHR Predictive Analytics

**DOI:** 10.1007/s41666-025-00222-8

**Published:** 2025-11-06

**Authors:** Chin Wang Cheong, Kejing Yin, William K. Cheung, Ivor Tsang

**Affiliations:** 1 Department of Computer Science, Hong Kong Baptist University, 224 Waterloo Road, Kowloon Tong, Hong Kong, China; 2 School of Computer Science, University of Technology Sydney, 15 Broadway Ultimo, Sydney, New South Wales 2007 Australia

**Keywords:** Explanability, Feature selection, Deep learning, Electronic health records, Predictive analytics

## Abstract

Identifying relevant input features which contribute to the output of a clinical prediction model can enhance the model explainability. To allow the explainability to be more personalized, instance-wise feature selection (IWFS) methods can be adopted where features are selected specifically for each input instance. Existing IWFS methods often grapple with feature selection instability, and thus precarious interpretation. As relevant features among the instances in a dataset do overlap, feature grouping tricks have been proposed to regularize the selection, but often at the expense of sacrificing the downstream prediction accuracy. To this end, we propose a novel instance-wise feature grouping method called FlexGPC to achieve robust and stable selection by learning i) flexible representation for feature groups, and ii) flexible combination of feature groups implemented using neural networks. To evaluate the effectiveness of FlexGPC, we explore various feature group combination schemes and conduct extensive experiments for performance comparison using real-world electronic health records (EHR) data. Our experimental results show that FlexGPC outperforms all the SOTA baselines in terms of accuracy and feature selection stability for both downstream mortality and next-admission diagnosis prediction tasks. We also illustrate that computational phenotyping can be achieved at the same time, with the identified feature groups being the potential phenotypes.

## Introduction

Machine learning (ML) methods have been found promising for clinical prediction tasks. Other than achieving high accuracy, their explanability is always emphasized due to the safety-critical nature of healthcare application and the increasing legal and ethical concerns of AI models [1–3]. Identifying relevant input features which contribute to the prediction outcome can allow clinicians to discover the key factors which lead to the outcome based on the ML model. This is essentially a *feature selection* problem which has been well studied in the literature [4, 5], and widely used for explaining relevant risk factors for different diseases [6–10]. For example, [9] adopted a bagging-based feature selection framework and identified smoking habits, lack of exercise, and unbalanced diet of both mothers and children to be the key risk factors of childhood obesity.

Conventional feature selection methods can only identify a common feature subset for all data instances. Yet there are many cases where the relevant feature subset varies over the instances in the dataset. For example, the relevant subset of clinical features for medical diagnosis should depend on the specific health condition of an individual patient. To support more personalized explanability, *instance-wise feature selection* (IWFS) methods can be adopted to identify the relevant feature subset per data instance [11, 12]. As the selected feature subset is considered relevant to the specific output of the downstream task, the IWFS model can be seen as an “explainer” that explains the prediction results through the selected features.

Despite the promising results, existing IWFS methods often grapple with feature selection instability, and thus precarious interpretation. It is always desirable for a feature selection method to select a consistent subset of features given reasonable variation of an input [13, 14]. Achieving that for IWFS, however, is challenging since a lot more selection variables are to be estimated. Neural network models (e.g., MLP) have been proposed to represent the selection mapping [11, 15–17]. Yet, learning the selection network often suffers from overfitting.

With the observation that relevant features for individual data instances in fact possess different degrees of overlapping, feature grouping tricks can be explored to regularize the feature selection to improve the selection stability. *Instance-wise feature grouping* (IWFG) methods have recently been proposed [16, 18] to carry out the instance-wise selection over a set of feature groups instead of individual features. In addition, the feature groups obtained via learning also form interpretable feature grouping patterns (e.g., phenotypes from EHR data), which have also been understood to be salient for the prediction task. Introducing the grouping for the gain in explanability and stability, however, often results in degradation of the downstream prediction accuracy due to the constraints imposed for the regularization.

**Fig. 1:**
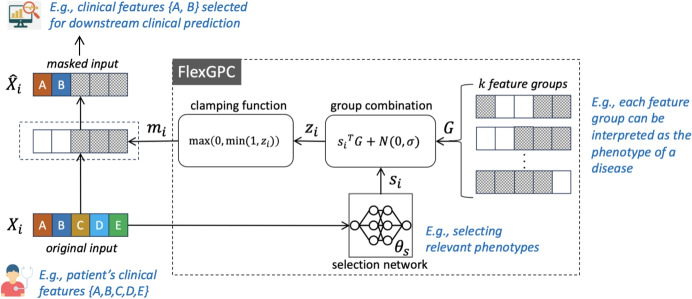
FlexGPC achieves instance-wise feature selection via adaptive feature group combination for enhancing clinical prediction model explanability. *G*, are the feature groups, FlexGPC combine them for each data point by the mixture weight *s*

To this end, we propose Flexible Group-wise Combination (FlexGPC) which aims to explain the prediction output by learning i) a set of more flexibly represented feature groups and ii) a selection network to achieve flexible combination (selection) of the feature groups. Compared with the existing IWFG methods, FlexGPC tries to “soften” the feature group representation can allow the relative importance of each feature within a feature group to be captured, and to “soften” their combination to open up more options for combining the feature groups to form the feature mask. The learning objective of FlexGPC is also designed to further emphasize the model interpretabilty. In this paper, two particular feature group combination schemes, namely *convex combination* and *restricted affine combination* are adopted and integrated into the proposed FlexGPC. The former is a natural choice while the latter enables a more flexible combination by allowing some features to be “de-selected”. Figure 1 illustrates the overall architecture of FlexGPC which can be incorporated into different model architectures for various clinical prediction tasks.

We conduct extensive experiments to evaluate the effectiveness of the proposed FlexGPC for mortality and next-admission diagnosis prediction tasks using the real-world EHR datasets. By properly relaxing the assumptions commonly used in existing IWFG methods, we demonstrate that FlexGPC can achieve substantial improvement in both prediction accuracy and selection stability over the SOTA baselines we tested. The improvement becomes more obvious when the data missingness rate is high which is common for EHR data. Among the two feature group combination schemes, restricted affine composition gives substantially better performance. We provide interpretation to the feature groups identified from the real-world EHR datasets. To illustrate its applicability to other problem domains, we also apply FlexGPC to image data and gene expression data to demonstrate its effectiveness. The key contributions of this paper can be summarized as follows:

1. We propose a novel IWFG model called FlexGPC that enables more flexible feature representation and grouping to achieve robust, stable and more explanable instant-wise feature selection.
2. We design the selection network to implement convex and restricted affine combinations for feature grouping in FlexGPC, and the corresponding learning algorithm to balance the objectives of flexibility of feature grouping and model interpretability.
3. We show that FlexGPC can be incorporated into different clinical analytics models for mortality and next-admission diagnosis prediction, with significant performance improvement over the SOTA IWFS methods in terms of both prediction accuracy and feature selection stability.

## Related Work

This section provides a brief review of methods proposed for instance-wise feature selection and grouping. With the objective of enhancing model explanability, a number of them were evaluated based on the EHR data analytics tasks.

### Instance-wise Feature Selection

Conventional feature selection aims at identifying a (*global*) relevant subset of features for the whole dataset. The problem has been well studied under different settings for the selection task, including supervised, semi-supervised, and unsupervised [19, 20]. *IWFS* tries to identify a distinct feature subset for each data instance. The selector-predictor approach is commonly adopted, in which a selector network is learned to map each input to a specific feature selection mask and the masked input is fed to a predictor network. For instance, L2X [11] performs instance-wise feature selection for explaining black-box models by maximizing the mutual information between the selected features and the response variable. INVASE [12] extends L2X so that the size of the feature subset selected can also be inferred based on the input instance. As the use of mutual information cannot capture causal influence [15], relative entropy distance together with sparse and class-discriminative features was adopted in [17]. Also, LLSPIN [21] was proposed for selecting features in low-sample-size data.

### Instance-wise Feature Grouping

Grouping highly correlated features is effective in reducing the feature selection solution space [22]. Existing methods for global feature grouping [23, 24] identify feature groups using clustering algorithms, and representative features per cluster can then be selected [25, 26]. This feature grouping idea has also been extended to the instance-wise setting. Instance-Wise Feature Grouping (IWFG) aims to identify feature groups from the data that can be specifically combined according to the input instance to form the feature mask. gI is an IWFG method that formulates the problem using two notions of feature redundancies based on information theory [16]. Additionally, GroupFS is a group-wise feature selection method proposed for supervised tasks [24], which first clusters the data instances and then identifies the cluster-specific feature subset.

### Feature Acquisition

Feature acquisition involves sequentially selecting a subset of features to achieve optimal prediction performance. [27] and [28] utilize Q-learning to sequentially select features. [29] propose a generative surrogate model that captures dependencies among input features to evaluate the potential information gained from the acquisitions. [30] propose an amortized optimization approach as an alternative to the reinforcement learning method, which is notoriously difficult to train. [31] select features in batches rather than individually to reduce query costs. In contrast to feature acquisition, instance-wise feature selection assumes all features are available upfront, allowing for improved accuracy.

To better situate FlexGPC within the literature, we provide Table 1 that summarize therepresentative instance-wise feature selection (IWFS) and instance-wise feature grouping (IWFG) approaches.

**Table 1:** Comparison of representative IWFS/IWFG methods

Method	Selector type	Grouping	Interpretability	Weaknesses
L2X	MLP	No	Salient features	Unstable, no grouping
INVASE	Actor–critic policy net	No	Adaptive subset size	High variance, unstable
LSPIN	Locally sparse NN	No	Sparse local masks	Misses global structure
gI	Info-theoretic redundancy	Yes	Learns groups	High complexity
GroupFS	MoE with discrete gating	Yes	Cluster-level subsets	Rigid, low flexibility

FlexGPC is designed to address their limitations by enhancing expressiveness (via RAC), stability (via regularization and clamping), and robustness under missingness

## Problem Formulation

### Key Challenges

While instance-wise feature selection (IWFS) enables more personalized and interpretable predictions, several fundamental challenges remain:

1. **Stability under perturbations.** IWFS models are highly sensitive to small variations in the input, which can lead to inconsistent feature subsets being selected for nearly identical instances. This instability reduces the reliability of the explanations in safety-critical domains such as healthcare.
2. **Accuracy–interpretability trade-off.** Introducing grouping or sparsity constraints improves interpretability but often comes at the cost of predictive accuracy. Balancing these objectives remains a central challenge in designing explainable selection models.
3. **Flexibility limits of convex mixing.** Existing IWFG methods that rely on convex combination of groups can only span a limited subset of possible feature masks. This lack of expressiveness prevents them from capturing more nuanced or counter-pattern structures that are needed in clinical settings.
4. **Robustness to missingness.** Real-world EHR datasets are plagued by high rates of missing values. A practical IWFS method must generate stable and meaningful masks even when large portions of the feature space are absent.

We denote $X \in \mathbb {R}^{n \times d}$ as the input data (e.g., clinical features) and $Y \in \mathbb {R}^{n}$ as the prediction labels (e.g., clinical outcomes), where *n* is the number of data instances (e.g., patient records) and *d* is the number of features. IWFS aims at learning a specific feature selection mask $m_i \in \mathbb {R}^{d}$ for each $i^{th}$ data instance $X_i \in \mathbb {R}^{d}$ where $0 \le m_{ij} \le 1$. The masked input $\hat{X_i}$ is obtained by taking the element-wise product $X_i\odot m_i$. The expected outcome of IWFS is to obtain $m_i$ for each $X_i$ so that the masked input $\hat{X_i}$ will correspond to the *relevant* features which in principle can give better prediction result than the case when the original input $X_i$ is used.

In this paper, we propose a novel IWFG method called FlexGPC which comprises a feature groups matrix and a selection network for estimating the values of the feature selection variables, as shown in Fig. 1. In the context of clinical prediction, the feature groups can be interpreted as phenotypes of different disorders. The role of the selection network is to select and combine the relevant phenotypes, and the masked input is the resulting set of relevant clinical features for the downstream prediction. Compared to the existing IWFG methods, FlexGPC allows soft feature groups and relax the assumptions typically imposed on selection variables (e.g., m-hot representation) for the feature grouping. In particular, we study two combination schemes, namely convex combination and restricted affine combination. A selection network can be designed to estimate the selection variables accordingly.

The proposed FlexGPC module can be integrated with a prediction model, and the overall model can be learned end-to-end (to be detailed in the sequel). We design our learning objective so that the masked input contains both informative and relevant features. Also, regularization terms are adopted to encourage sparsity for both the feature mask and the feature groups to enhance the interpretability of FlexGPC.

### Overall Framework of FlexGPC

We first describe two key components of FlexGPC: i) feature groups and ii) feature group selection network for implementing different feature group combination schemes.

#### Feature Groups

Let $G \in \mathbb {R}^{k \times d}$ represent a set of feature groups, where *k* is the number of groups and *d* is the number of features. The elements of *G* can take values in the range of $0 \le G_{ij} \le 1$. $G_j \in \mathbb {R}^{d}$ is a vector representing the $j^{th}$ feature group with its elements indicating the feature importance within the group.

#### Feature Group Combination

Let $s_i \in \mathbb {R}^k$ be a vector of selection variables indicating the extent that the *k* different feature groups should be selected for the data instance $X_i$. The value of $s_i$ is computed by feeding $X_i$ into a selection network which is to be learned. Instead of assuming m-hot representation for $s_i$ as in [16], we allow $s_{ij}$ to take continuous values. The “selection” step essentially becomes an input-specific combination process. In the sequel, we will call the selection variables as *selection weights*.

In particular, we investigate two combination schemes for representing a feature mask, namely *convex* combination, and *restricted affine* combination.

##### Definition 1

Convex Combination (CC) aggregates a set of feature groups $G_1, G_2, \ldots , G_k$ for an input $X_i$ with the corresponding selection weights $s_{i1}, s_{i2}, \ldots , s_{ik}$ where $\sum _j s_{ij} = 1$ and $0 \le s_{ij} \le 1$ for all $j=1,...k$.

The value of $s_i$ is computed by feeding $X_i$ to a selection network designed accordingly. Instead of using multi-hot representation, we allow $0 \le s_{ij} \le 1$ for $j=1,...k$. Thus, the selection network is essentially performing an input-specific *convex* combination process. We can also explore other ways of combination.

##### Definition 2

Restricted Affine Combination (RAC) aggregates a set of feature groups $G_1, G_2, \ldots , G_k$ for an input $X_i$ via a weighted sum with the corresponding weights $s_{i1}, s_{i2}, \ldots , s_{ik}$, where $\sum _j |s_{ij}| = 1$ and $-1 \le s_{ij} \le 1$ for all $j=1,...k$.

RAC deliberately allows the selection weights computed by the selection network to take negative values. This means that we allow one feature group to *de-select* its associated features which are selected due to other feature groups.

**Fig. 2:**
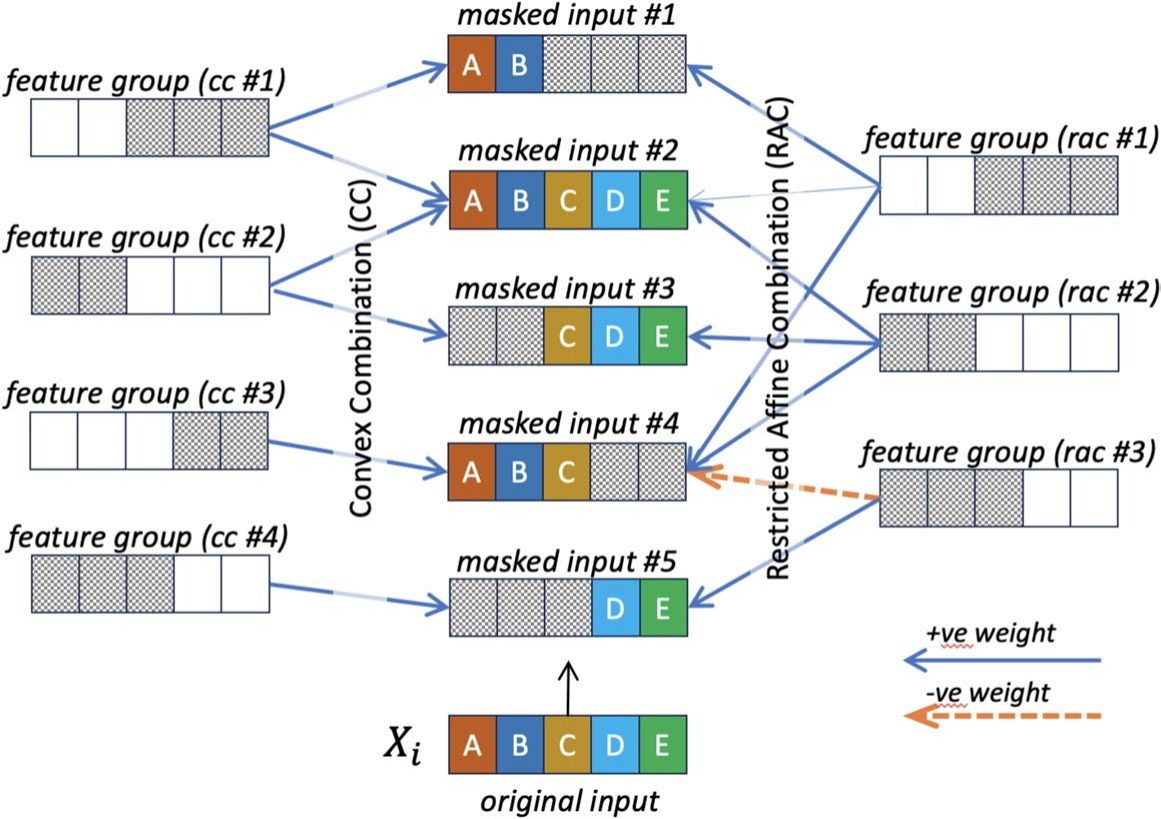
Illustration of masked inputs which require 4 feature groups based on CC (left) but only 3 groups using RAC by allowing negative selection weight (right)

Figure 2 illustrates the potential benefit of introducing feature de-selection in the combination process. If only positive values are allowed for the selection weights, we need four feature groups to represent the five hypothetical masked inputs. If negative selection weights are allowed, only three feature groups are needed. In general, given a fixed number of feature groups, RAC can allow more feature masks to be represented compared to CC. It is not difficult to theoretically show that RAC can span a larger subspace of feature masks than CC.

##### Theorem 1

**(Expressiveness of Restricted Affine Composition)**. Suppose $d>$1, $k_1<d$, for any space spanned by a matrix $G_1 \in R^{k_1 \times d}$ with convex composition, that exist $G_2 \in R^{k_2 \times d}$ span the larger or equal space with restricted affine composition, and $k_2 < k_1$, if the following hold true:

*Condition:* There is a feature group separation of $G_1$ to $G_{1,1:k_2}$ and $G_{1, k_2:k_1}$ such that $G_{1, k_2:k_1}$ are pairwise differences of $G_{1,1:k_2}$.

##### Proof

The space spanned by $G_1$ is a subspace of $\mathbb {R}^d$, we will denote it by $S_{G_1}$. By the feature group separation condition, we can write any vector $\textbf{v}$ in $S_{G_1}$ as a convex combination of the vectors in $G_{1,1:k_2}$ and the pairwise differences in $G_{1, k_2:k_1}$. We also denote $G_2=G_{1,1:k_2}$, and we denote the space spanned by $G_2$ by $S_{G_2}$.

We can express any vector $\mathbf {m_i}$ in the space spanned by $G{1, k_2:k_1}$, as a restricted affine combination of the vectors in $G_{2}$. This is due to the condition that $G_{1, k_2:k_1}$ are pairwise differences of $G_{1,1:k_2}$, and we know that restricted affine composition allows for negative weights, which can express differences between vectors.

Furthermore, any vector in $G_{1,1:k_2}$ can also be expressed as a restricted affine combination of vectors in $G_{2}$ as they are the same, by choosing the weights such that they sum up to one and no weight is less than zero, effectively creating a convex combination.

So, any vector in the space spanned by $G_1$ can be expressed as a restricted affine combination of the vectors in $G_2$. Therefore, $S_{G_1} \subseteq S_{G_2}$. $\square$

##### Feature Mask *m*

For each visit *i*, we compute the feature mask using restricted affine composition $m_i =U( s_1 \textbf{g}_1 + s_2 \textbf{g}_2 + \ldots + s_n \textbf{g}_n)$. Where $g_i$ is the ith feature group of *G* and $0 \le m_i \le 1$. And *U*(*w*) is a clamping function that map *w* from 0 to 1:$U(w)=max(0,min(1,w))$.

We could define the *clamped restricted affine composition*(CRAC) as *U*(*s*) where *w* is defined in the definition 2. Similarly, we define *clamped convex composition* as *U*(*s*) where *s* is defined in the definition 1.

Clamped restricted affine composition is more expressive than restricted affine Composition when $0 \le G \le 1$. As shown below:

##### Theorem 2

**(Expressiveness of Clamped Restricted Affine Composition)**. we denote the space spanned by *G* with restricted affine composition as $S_{G}$, and the space spanned by *G* with clamped restricted affine composition as $\hat{S_{G}}$. And $G>=0$. $S_{G} \subset \hat{S_{G}}$ if some feature groups overlap.

Where we define two feature groups $g_1$ and $g_2$ are overlap If $\exists j \in \mathbb {D}, g_{1j},g_{2j}>0$ and $\exists j \in \mathbb {D}, g_{1j}\ne g_{2j}$

##### Proof

For any vector *v* in $S_{G}$, we can construct it using clamped restricted affine composition with exactly the same set of $s_1, s_2, \ldots , s_n$. as for $\forall j\in \mathbb {D}$, $0 \le v_j \le 1$. So the clamp function *U*(.) have no effect on it. Therefore, $S_{G} \subseteq \hat{S_{G}}$.

Then, we show how to construct *v* in $\hat{S_{G}}$ but not in $S_{G}$. Suppose $g_1$ and $g_2$ are two feature groups that are overlapped; we calculate the difference between them as $g_3=g_1-g_2$. $g_3$ cannot be constructed by restricted affine composition as $\exists j \in \mathbb {D}, g_{3j}<0$, which violate the positivity of G. With *U*(.), we can map the negative value to 0, so $g_3 \in \hat{S_{G}}$. Thus, $S_{G} \subset \hat{S_{G}}$

Apart from expressiveness, stability of learned masks is a important criteria that make sure the differences of masks learned during different runs are small. In the following, we will show some definition and properties of the stability and showing stability of the proposed method.

##### Definition: Uniform Stability

A method is said to be $\beta$-uniformly stable if, for any two datasets *S* and $S'$ that differ by one point, and for any input $x_i$, the computed feature masks $m_i$ and $m_i'$ from *S* and $S'$ respectively differ by no more than $\beta$.

Mathematically, this can be expressed as:

$$\Vert m_i - m_i' \Vert _2 \le \beta$$

for all $x_i$ in the input space, where $m_i = f(S, x_i)$, $m_i' = f(S', x_i)$, *f* is the method being used (in this case, the feature mask computation using composed feature grouping), and $\Vert \cdot \Vert _2$ is the Euclidean norm. The constant $\beta$ represents the maximum allowed change in the feature mask due to a single change in the dataset.

##### Assumption 1

**Feature Group Learning Stability**

Suppose we have two datasets *S* and $S'$ that differ by one point. Let *G* and $G'$ be the feature groups learned from *S* and $S'$ respectively. We assume that the maximum difference between corresponding feature groups in *G* and $G'$ is bounded by a constant $\gamma$.

$$\max _{i \in \{1,...,k\}} \Vert G_{i} - G'_{i} \Vert _2 \le \gamma$$

Here, $G_{i}$ and $G'_{i}$ represent the *i*-th feature group in *G* and $G'$ respectively, $\Vert \cdot \Vert _2$ is the Euclidean norm, and $\gamma$ is a constant representing the maximum change in the feature groups due to a single change in the data. This assumption essentially states that the feature group learning method is stable, in the sense that a small change in the data results in at most a $\gamma$-sized change in the feature groups.

##### Property: Lipschitz Continuity of the Restricted Affine Composition

The restricted affine composition of a set of vectors $\textbf{v}_1, \textbf{v}_2, \ldots , \textbf{v}_n$ with corresponding scalar weights $s_1, s_2, \ldots , s_n$ is a Lipschitz continuous function due to its linearity.

This is mathematically expressed as follows:

$$\Vert w(\textbf{v}) - w(\textbf{v}') \Vert _2 \le L \Vert \textbf{v} - \textbf{v}' \Vert _2$$

for all vectors $\textbf{v}$ and $\textbf{v}'$, where $w(\textbf{v}) = s_1 \textbf{v}_1 + s_2 \textbf{v}_2 + \ldots + s_n \textbf{v}_n$, *L* is the Lipschitz constant, and $\Vert \cdot \Vert _2$ is the Euclidean norm.

Given the constraints on the weights ($|s_1| + |s_2| + \ldots + |s_n| = 1$ and $-1 \le s_1, s_2, \ldots , s_n \le 1$), the Lipschitz constant for this operation is 1, meaning that this operation is 1-Lipschitz continuous. Therefore, the distances between points in the input space are not increased by this operation.

##### Theorem 2

(Uniform Stability of the Method)

Suppose we have two datasets *S* and $S'$ that differ by one point, and corresponding feature groups *G* and $G'$ such that $\Vert G - G' \Vert _2 \le \gamma$. Let $f(S, x_i)$ and $f(S', x_i)$ be the feature masks computed for any input $x_i$ under datasets *S* and $S'$ respectively, where *f* represents our method. Let the Lipschitz constant of the restricted affine composition used in the method be *L*.

Then, the method is $L\gamma$-uniformly stable, i.e., for any input $x_i$, the difference between the computed feature masks is bounded by $L\gamma$:

$$\Vert f(S, x_i) - f(S', x_i) \Vert _2 \le L \gamma$$

The above inequality means that a small change in the input data (at most one data point) leads to a bounded change in the computed feature masks, thus demonstrating the stability of the method.

##### Proof

We start with two datasets *S* and $S'$, which differ by at most one point. From assumption 1, we have the stability of feature groups learning, which implies that $| G - G' |_2 \le \gamma$ for corresponding feature groups *G* and $G'$.

Now, let’s consider an input $x_i$ for which we compute the feature masks $m_i = f(S, x_i)$ and $m_i' = f(S', x_i)$ using datasets *S* and $S'$, respectively.

Using the Lipschitz continuity of the restricted affine composition (property), the difference in the computed feature masks is bounded by the Lipschitz constant multiplied by the difference in the feature groups, i.e., $| m_i - m_i' |_2 \le L | G - G' |_2$.

Substituting the upper bound of $| G - G' |_2$ from assumption 1 into this inequality, we have $| m_i - m_i' |_2 \le L \gamma$.

Thus, we have shown that for any input $x_i$, the difference between the computed feature masks $m_i$ and $m_i'$ is bounded by $L\gamma$, which completes the proof of $L\gamma$-uniform stability of the method.

#### Selection Network Implementation

We implement the selection network using MLP. For CC, the selection network, denoted as $\theta ^{cc}_s(X_i)$, computes the output $s_i \in \mathbb {R}^k$ using the function:

1 $$\begin{aligned} s_i~=~\theta _s^{cc} (X_i)~=~{\text {softmax}}\left( W_2 {\text {ReLu}}(W_1 X_i)\right) \end{aligned}$$

where $W_1 \in \mathbb {R}^{l \times d}$ and $W_2 \in \mathbb {R}^{k \times l}$ are the parameters of the selection network. The softmax function is used to guarantee $0 \le s_{ij} \le 1$ for all *j* and $\sum _j s_{ij} =1$.

For RAC, the selection network, denoted as $\theta ^{rac}_s(X_i)$, computes the output $s_i \in \mathbb {R}^k$ using the function:

2 $$\begin{aligned} s_i~=~\theta ^{rac}_s(X_i)~=~\frac{W'_2 {\text {ReLu}}(W'_1 X_i)}{ || W'_2 {\text {ReLu}}(W'_1 X_i) ||_1} \end{aligned}$$

where $W'_1 \in \mathbb {R}^{l \times d}$ and $W'_2 \in \mathbb {R}^{k \times l}$ are the parameters of the selection network $\theta ^{rac}_s()$, and $\Vert | \cdot \Vert |_1$ denotes the L1-norm. The use of the formula can guarantee $-1 \le s_{ij} \le 1$ for all *j*, and $\sum _j |s_{ij}| =1$.

#### Feature Mask via Groups Selection

Finally, the feature mask $m_i$ for $X_i$ can be computed as $m_i = s_i^TG$. To achieve a more robust mask, we adopt the LSPIN mapping [21] during the model training:

3 $$\begin{aligned} m_i = {\text {max}}(0,{\text {min}}(1, s_i^TG +\epsilon )) \end{aligned}$$

where $\epsilon$ is drawn from $N(0,\sigma )$ during model training, and $\sigma$ is a hyperparameter for setting the noise level. The mapping has the benefit of pushing the elements in $m_i$ to take values closer to either 0 or 1. Adding noises helps the model explore more combinations of feature groups during training, which can in turn improve the model robustness. After training, the mask can be computed using (3) by setting $\epsilon$ to zero.

**Fig. 3:**
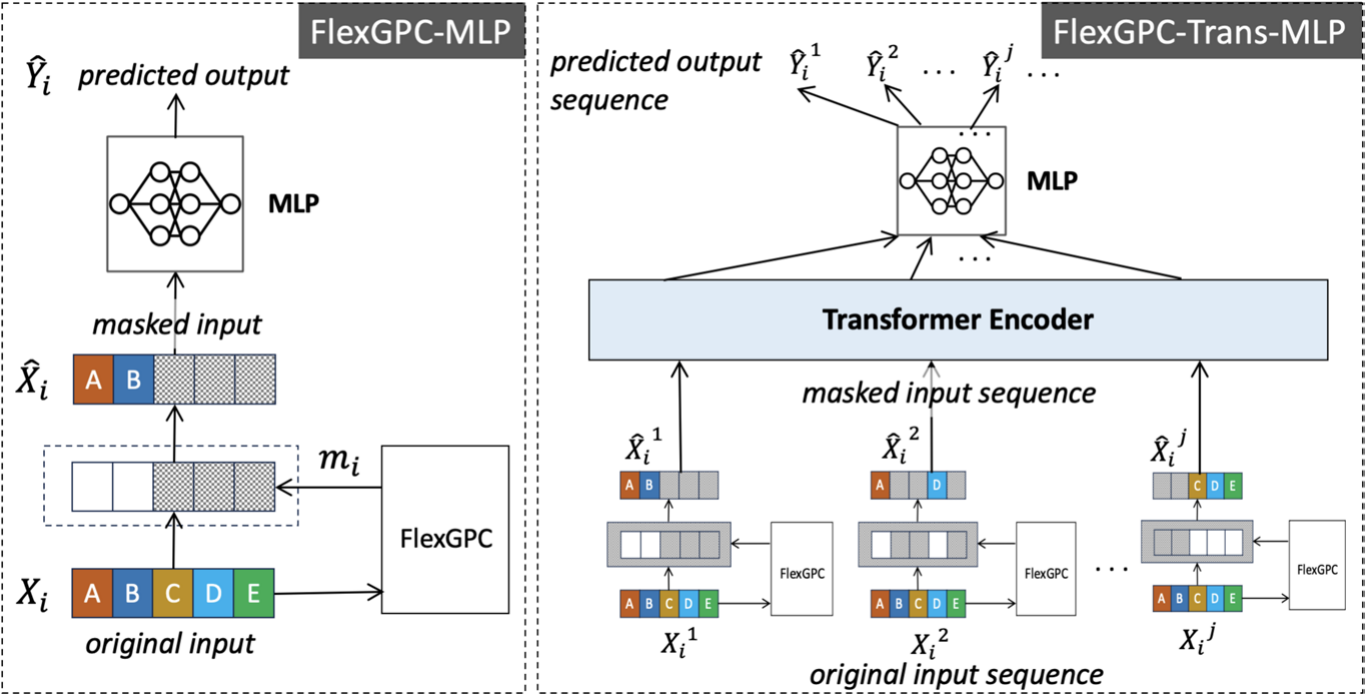
FlexGPC incorporated into classification and sequence-to-sequence prediction models

### Prediction Models with FlexGPC Incorporated

FlexGPC can be incorporated into different prediction models and train them end-to-end to achieve robust and stable instance-wise feature grouping and selection, as well as high prediction accuracy. In particular, we apply FlexGPC to two prediction tasks: mortality prediction and next-admission diagnosis prediction. Depending on whether the sequential relationship of the features are exploited or not, the two tasks can readily be formulated as sequence-to-sequence classification or just standard classification.

For standard classification, as shown in Fig. 3, we can first feed the input feature vector to FlexGPC and then the masked input to an MLP to form *FlexGPC-MLP*. For sequence-to-sequence classification, we can integrate FlexGPC with sequential models like Transformer. Specifically, given an input sequence $\textbf{X}_i=(X_i^1, X_i^2,..., X_i^{Li})$ where $X_i^j \in \mathbb {R}^d$ and the corresponding output label sequence $\textbf{Y}_i=(Y_i^1, Y_i^2,..., Y_i^{Li})$ where $Y_i^j \in \mathbb {R}$, we can feed each element $X_i^j$ to FlexGPC to obtain its masked version $\hat{\textbf{X}}_i^j$. Then, we feed the masked input sequence $\hat{\textbf{X}}_i$ to a Transformer, and then to an MLP to form *FlexGPC-Trans-MLP*.

#### Model Training

Both FlexGPC-MLP and FlexGPC-Trans-MLP can be trained end-to-end to achieve good feature selection and prediction performance at the same time. For the training, we adopt the following objective function with three loss terms:

4 $$\begin{aligned} \mathcal {L}=\mathcal {L}_{\text {pred}}+\mathcal {L_{\text {recons}}}+\mathcal {L}_{\text {reg}}. \end{aligned}$$

*a) Prediction Loss* is measured by cross-entropy:

5 $$\begin{aligned} \mathcal {L}_{\text {pred}} = -\frac{1}{n} \sum ^{n}_{i=1} \left[ \textbf{Y}_{i}^\intercal \log (\hat{\textbf{Y}}_{i}) + (1-\textbf{Y}_{i})^\intercal \log (1-\hat{\textbf{Y}}_{i}) \right] . \end{aligned}$$

to guide the model learning to give high prediction accuracy given the *relevant* features selected.

*b) Reconstruction Loss* measures the discrepancy between $\textbf{X}_i$ and its reconstructed version based on the masked input $\hat{\textbf{X}}_{\textbf{i}}$:

6 $$\begin{aligned} \mathcal {L}_{\text {recons}}=\sum ^n_{i=1} || X_i-\phi (\hat{X_i})|| \end{aligned}$$

where $\phi ()$ is implemented using a two-layer MLP. It encourages the *informative* features that could recover the redundant features to be captured and retained.

*c) Regularization Term* comprises two parts:

7 $$\begin{aligned} \mathcal {L}_{\text {reg}} = \mathcal {L}^m_{\text {reg}} + \mathcal {L}^G_{\text {reg}} = \sum _{i,j} m_{ij} + I(G) \end{aligned}$$

$$\begin{aligned} I(G) = - \frac{1}{d} \sum _{i=1}^{\text {d}} \sum _{j=1}^{\text {k}} G^T_{ij} \log (G^T_{ij}) - \frac{1}{k} \sum _{i=1}^{\text {k}} \sum _{j=1}^{\text {d}} G_{ij} \log (G_{ij}) \end{aligned}$$

where $\mathcal {L}^m_{\text {reg}}$ encourages sparse mask, and $\mathcal {L}^G_{\text {reg}}$ encourages discriminative and sparse feature groups. Both are introduced for enhancing FlexGPC’s interpretability.

Note that before training directly on $\mathcal {L}$, we pre-train the feature masks $m$ based on the following *pre-training loss*:

8 $$\begin{aligned} \mathcal {L}_{\text {pretrain}} = \sum _{i,j} (m_{ij} - \textbf{X}_{i,j})^2. \end{aligned}$$

We find that using this loss for pre-training can force the feature masks $m$ to be similar to the input $\textbf{X}$. It allows the parameters of the selection network to be initialized less randomly before the subsequent model training, thereby further improving the selection stability.

**Table 2:** Statistics of datasets

Data set	MIMIC-III	eICU	Gene	MNIST
# of Samples	6,453	12,293	1,937	60,000
# of Features	6,054	3,353	20,125	784
Average visits per patients	2.7	2.2	/	/

## Experiment Setup

### Datasets

To evaluate the effectiveness of FlexGPC for clinical prediction tasks, two real-world EHR datasets, namely MIMIC-III (Medical Information Mart for Intensive Care) [32] and eICU [33], are used.

MIMIC-III is a public dataset containing data of over 46,000 patients admitted to intensive care units (ICU). eICU is a multi-center database, containing over 200,000 ICU admissions. We filter out patients with less than 2 hospital admissions to allow next-diagnosis prediction to be carried out. As a result, we extract 6, 453 patients with 2.7 admissions per patient on average for MIMIC-III, and 12, 293 patients with 2.2 admissions on average for eICU. The average numbers of diagnoses and medications per admission are 12.0 and 38.9 respectively for MIMIC-III, while 14.6 and 21.4 for eICU.

To further demonstrate that FlexGPC is also applicable to other problem domains, we conduct additional experiments using a gene expression dataset and the MNIST image dataset. For the gene expression dataset, we use one that contains cells collected from the human pancreas, with 1,937 cells, 20,125 genes, and 14 cell types.^1^ We follow the pre-processing adopted in [34], where genes expressed in less than three cells are excluded from further analysis and the gene expression counts per cell are normalized. MNIST is a database of handwritten digits, which contains 60,000 training images. Each image is a 28x28 pixel grayscale image of a handwritten digit (0 through 9). And there are 10,000 test images.

The statistics of the datasets are summarized in Table 2.

### Parameter Setting

For implementing $\theta _s^{cc}()$, $\theta _s^{rac}()$ and $\phi ()$, the size of the hidden layer *l* is 400. For the prediction MLP in FlexGPC-MLP and FlexGPC-Trans-MLP, the size of the hidden layer is 200. The Transformer used in FlexGPC-Trans-MLP has one layer, a single head and a dimension of 200. We tested different numbers of feature groups *k* from 50 to 300. For model training, $\sigma$ in (3) is set to 1. The batch size is 100. $80\%$ of the data is used for training, $10\%$ for validation, and $10\%$ for testing. We run our experiments on a server with four NVidia Tesla V100-PCIE-32GB GPU, 250 GB memory, and Intel(R) Silver 4114 CPU. Adam optimizer is used for the training with five repetitions.

### Performance Evaluation

We test the performance of FlexGPC first on two clinical prediction tasks: i) next-admission diagnosis prediction [35], and ii) mortality prediction [36]. They are formulated as sequence-to-sequence classification problems with FlexGPC-Trans-MLP adopted. We further test the effectiveness of FlexFPC on two other problems iii) cell type identification [34], and iv) handwritten digit recognition. They are formulated as standard classification problems where FlexGPC-MLP is adopted.

#### Metrics for Prediction Accuracy

For the next-admission diagnosis prediction [37, 38], we first derive the ground-truth labels by grouping the diagnoses in the next admissions into 793 groups based on the first three digits of their ICD-9 codes, and then carry out multi-label classification accordingly. The prediction accuracy is measured by:

$$\begin{aligned} Accuracy@k=\frac{\text {\# of true positives in the top }k\text { predictions}}{\text {\# of positives}}. \end{aligned}$$

For mortality prediction, we use *area under the ROC curve (AUC)*. For cell type identification and handwritten digita recognition, we use *Accuracy@1*.

#### Metric for Feature Selection Stability

We evaluate the stability of feature selection by:

$$\begin{aligned} Stability(i)=\frac{\sum _{p,q \in B}RankSim(m_i^p,m_i^q)}{B^2} \end{aligned}$$

It measures the similarity of the feature masks learned in *B* runs (5 in our experiments), where

$$\begin{aligned} RankSim(m_i^p, m_i^q) = \frac{\sum _{i=1}^d\mathbbm {1}\{ \text {index}_i(m_i^p) = \text {index}_i(m_i^q) \}}{d} \end{aligned}$$

gives the ranking similarity of the two masks $m_i^p$ and $m_i^q$ obtained in $p^{th}$ and $q^{th}$ runs respectively. The higher the value, the better the stability is.

### Baselines

We compare the performance of FlexGPC-Trans-MLP and FlexGPC-MLP with a number of baselines.

- *INVASE* [12] consists of a selector network, a predictor network, and a baseline network and uses the actor-critic methodology for training.
- *LSPIN* [21] is a locally sparse neural network where the local sparsity is learned to identify the relevant feature subset for each data instance.
- *gI* [16] learns and combine feature groups to form the feature mask by minimizing the loss of representation and relevant redundancies.
- *GroupFS* [24] is a group-wise feature selection method that uses the Mixture of Experts (MoE) model with discrete gating to select feature masks.
- $\text {FlexGPC}_{CC}$ replaces the restricted affine combination (RAC) component of FlexGPC with the convex combination (CC).

For fair comparison, we modify all the baselines so that they adopt the same prediction network as in FlexGPC-Trans-MLP or FlexGPC-MLP, depending on the prediction task.

For the two sequence-to-sequence prediction tasks, we report also the performance of *Hi-BEHRT* [39] which adopts a more sophisticated hierarchical Transformer to capture information of long sequences in the EHR.

## Results

This section presents the results of performance comparison with the baselines based on the four prediction tasks.

**Table 3:** Performance comparison based on MIMIC-III

*missing rate*			
**Model**	**0%**	**20%**	**40%**
Next-admission diagnosis prediction (Accuracy@20)			
INVASE	0.571 ± 0.015	0.609 ± 0.019	0.589 ± 0.017
LSPIN	0.615± 0.022	0.600 ± 0.023	0.598 ± 0.025
gI	0.602 ± 0.014	0.600 ± 0.017	0.591 ± 0.018
GroupFS	0.592 ± 0.012	0.589 ± 0.012	0.586 ± 0.014
$\text {FlexGPC}_{CC}$	0.610 ± 0.019	0.592 ± 0.019	0.586 ± 0.018
$\text {FlexGPC}_{Avg}$	0.589 ± 0.011	0.576 ± 0.016	0.571 ± 0.012
$\text {FlexGPC}_{MLP}$	0.575 ± 0.09	0.572 ± 0.011	0.561 ± 0.015
$\text {FlexGPC}$	**0.646± 0.016**	**0.621 ± 0.015**	**0.614 ± 0.017**
Hi-BEHRT	0.631 ± 0.013	0.618± 0.012	0.610 ± 0.011
Mortality prediction (AUC)			
INVASE	0.872 ± 0.021	0.839 ± 0.019	0.812 ± 0.020
LSPIN	0.944 ± 0.010	**0.880 ± 0.015**	0.877 ± 0.016
gI	0.917 ± 0.014	0.835 ± 0.014	0.810 ± 0.017
GroupFS	0.845 ± 0.011	0.837 ± 0.013	0.823 ± 0.013
$\text {FlexGPC}_{CC}$	0.859 ± 0.008	0.848 ± 0.010	0.836 ± 0.012
$\text {FlexGPC}_{Avg}$	0.896 ± 0.015	0.892 ± 0.017	0.881 ± 0.014
$\text {FlexGPC}_{MLP}$	0.862 ± 0.017	0.855 ± 0.022	0.853 ± 0.013
$\text {FlexGPC}$	**0.945 ± 0.010**	0.879 ± 0.012	**0.878 ± 0.011**
Hi-BEHRT	0.865 ± 0.021	0.832 ± 0.025	0.824 ± 0.023

FlexGPC-Trans-MLP is abbreviated as FlexGPC

**Table 4:** Performance comparison based on eICU

*missing rate*			
**Model**	**0%**	**20%**	**40%**
Next-admission diagnosis prediction (Accuracy@20)			
INVASE	**0.900± 0.011**	0.878 ± 0.012	0.856 ± 0.015
LSPIN	0.885 ± 0.009	0.876 ± 0.010	0.854 ± 0.010
gI	0.882 ± 0.007	0.879 ± 0.07	0.856 ± 0.09
GroupFS	0.870 ± 0.010	0.862 ± 0.012	0.835 ± 0.011
$\text {FlexGPC}_{CC}$	0.873± 0.007	0.863 ± 0.008	0.845 ± 0.100
$\text {FlexGPC}_{Avg}$	0.869± 0.006	0.872 ± 0.009	0.849 ± 0.8
$\text {FlexGPC}_{MLP}$	0.865± 0.008	0.860 ± 0.007	0.841 ± 0.13
$\text {FlexGPC}$	0.896± 0.009	**0.882± 0.010**	**0.863± 0.012**
Hi-BEHRT	0.872± 0.019	0.867 ± 0.017	0.842 ± 0.018
Mortality prediction (AUC)			
INVASE	0.731 ± 0.015	0.710 ± 0.014	0.703 ± 0.016
LSPIN	0.719 ± 0.012	0.701 ± 0.013	0.695 ± 0.013
gI	0.712 ± 0.015	0.710 ± 0.017	0.687± 0.018
GroupFS	0.638 ± 0.012	0.617 ± 0.012	0.552 ± 0.015
$\text {FlexGPC}_{CC}$	0.736± 0.007	0.731 ± 0.008	0.687± 0.011
$\text {FlexGPC}_{Avg}$	0.738± 0.012	0.725 ± 0.009	0.682± 0.013
$\text {FlexGPC}_{MLP}$	0.722± 0.005	0.724 ± 0.007	0.667± 0.09
$\text {FlexGPC}$	**0.740± 0.006**	**0.740± 0.010**	**0.729± 0.011**
Hi-BEHRT	0.716 ± 0.018	0.695 ± 0.021	0.695 ± 0.019

**Fig. 4:**
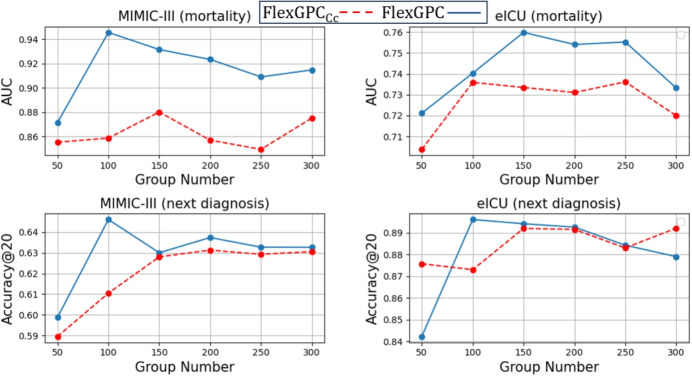
Effect of the number of feature groups on the accuracy of $\text {FlexGPC}_{CC}$ (red dashed line) and $\text {FlexGPC}$ (blue line). $\text {FlexGPC}$ outperforms $\text {FlexGPC}_{CC}$ in most cases

### Performance on EHR Data

The performance comparison results on next-admission diagnosis prediction and mortality prediction are summarized in Table 3 (MIMIC-III) and Table 4 (eICU). We observe that the proposed FlexGPC generally outperforms baselines under different degrees of data missingness. Using RAC for combining the feature groups in most cases outperforms the use of CC. This shows the benefit of RAC to allow the possibility of learning feature groups for “de-selection”. Compared with Hi-BEHRT which adopts a more sophisticated Transformer, $\text {FlexGPC}$ together with only a simple Transformer obtains better performance. Also, Fig. 4 shows that for next-admission diagnosis prediction, $\text {FlexGPC}$ consistently outperforms $\text {FlexGPC}_{CC}$ for different number of feature groups being tested. The same results are observed for mortality prediction. In general, FlexGPC which uses the RAC for the feature groups combination requires a smaller number of feature groups to achieve higher accuracy as compared to $\text {FlexGPC}_{CC}$.

**Table 5:** Stability comparison for MIMIC-III for next-admission diagnosis prediction

Model/Noise level	0	0.1	0.5
INVASE	0.815	0.801	0.785
LSPIN	0.809	0.792	0.773
gI	0.835	0.817	0.799
GroupFS	0.826	0.811	0.794
$\text {FlexGPC}_{CC}$	0.878	0.843	**0.806**
$\text {FlexGPC}$	**0.882**	**0.852**	0.795

To further confirm the effectiveness of CC and RAC for feature grouping, we test two variants of FlexGPC for selecting features and combining feature groups. $\text {FlexGPC}_{Avg}$ aggregates the feature groups via simple averaging, while $\text {FlexGPC}_{MLP}$ learns the feature mask using a one-layer neural network without feature grouping. As shown in Tables 3 and 4, $\text {FlexGPC}$ can outperform both by a large margin. For example, for the next-admission diagnosis prediction, the Accuracy@20 on MIMIC-III drops from 0.646 for $\text {FlexGPC}$ to 0.589 for $\text {FlexGPC}_{Avg}$ and 0.575 for $\text {FlexGPC}_{MLP}$.

Regarding feature selection stability, Table 5 shows the stability scores of $\text {FlexGPC}$ and $\text {FlexGPC}_{CC}$ for next-admission diagnosis prediction. To test also their robustness, we conduct the test with different levels of Gaussian noise added to the input. Both outperform all the baselines. gI is the second best where feature grouping is also adopted. Similar improvement is also observed on eICU data, and for mortality prediction.

**Fig. 5:**
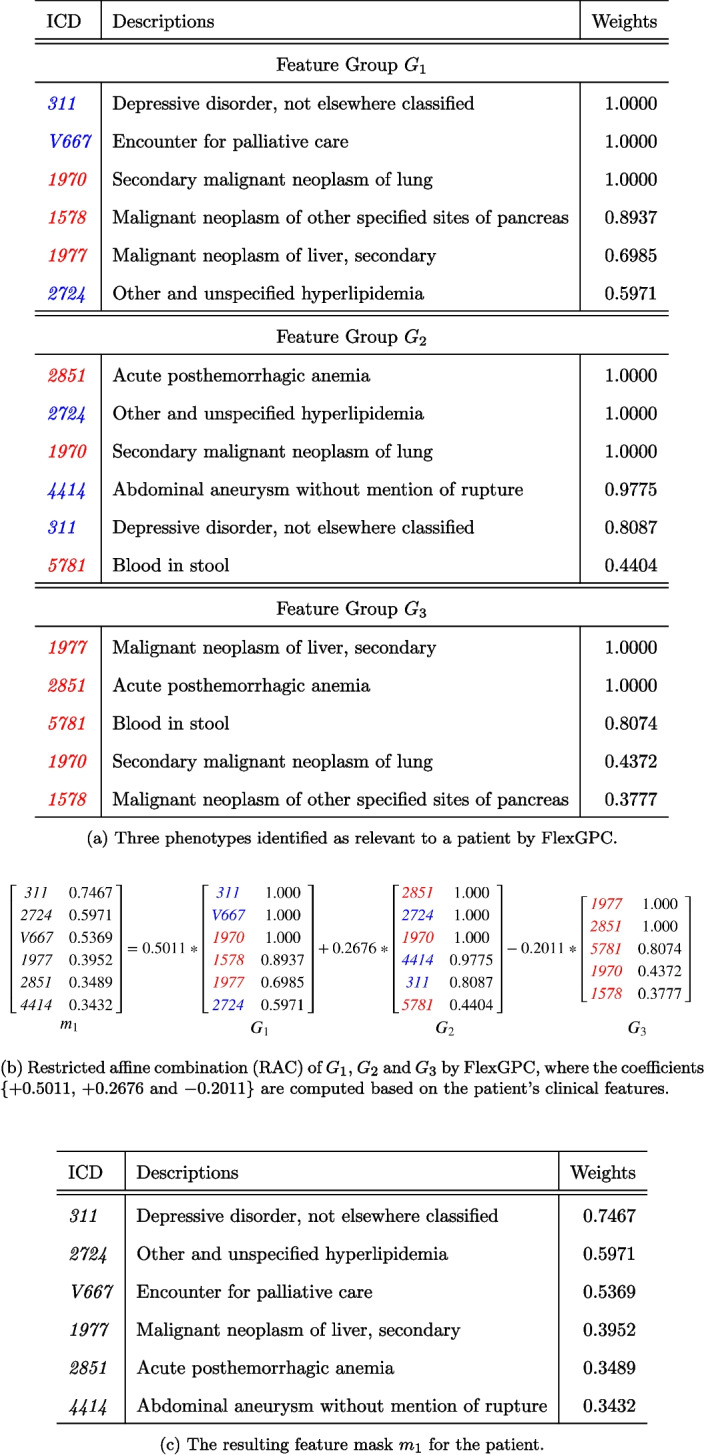
Illustration of a feature mask obtained by $\text {FlexGPC}$ for a patient record in MIMIC-III

### Phenotypes Extracted from MIMIC-III

Figure 5 shows three feature groups (phenotypes) ($G_1$, $G_2$, $G_3$) extracted from MIMIC-III using FlexGPC. With reference to a specific patient, $G_1$ and $G_2$ are identified by FlexGPC as positive (selected) feature groups, and $G_3$ as a negative (deselected) group. Figure 5a illustrates how the three feature groups can be combined using RAC estimated by the selection network to give a feature mask shown in Fig. 5b ).

The feature mask suggests that the patient is dealing with a serious, potentially metastatic cancer, accompanied by psychological (depression) and metabolic (hyperlipidemia) comorbidities and a high-risk vascular condition (aneurysm). $G_1$ is a group consisting of mental health issues that are related to the patients (*311*, *V667*) and some neoplasms-related diseases. However, not all diseases in $G_1$ are in the patient’s record. E.g., the patient did not develop *1578* (Malignant neoplasm of other specified sites of pancreas) and *1970* (Secondary malignant neoplasm of lung). The model can de-select them in $G_1$ by subtracting from it feature group $G_3$ which contains those diseases. Similarly, $G_2$ is related to bleeding issues which contain diseases related to the patient (*2724*, *4414*, *311*). Those unrelated diseases (*2851*, *5781*) again can be de-selected by subtracting from it $G_3$. The capability of identifying the feature groups which can be combined via group selection and de-selection to represent the whole dataset is a key feature enabled by FlexGPC.

**Table 6:** Accuracy and stability comparison on human pancreas gene expression and MNIST datasets

	Cell Type Identification		Handwritten Digit Recognition	
**Model**	Accuracy@1	Stability	Accuracy@1	Stability
INVASE	0.948 ± 0.012	0.831	0.977 ± 0.090	0.810
LSPIN	0.943 ± 0.010	0.829	0.963 ± 0.011	0.833
gI	0.747 ± 0.013	0.861	0.965 ± 0.012	0.857
GroupFS	0.769 ± 0.013	0.881	0.958 ± 0.015	0.823
$\text {FlexGPC}_{CC}$	0.943 ± 0.011	0.872	0.963 ± 0.011	0.866
$\text {FlexGPC}$	**0.959 ± 0.012**	**0.899**	**0.979 ± 0.011**	**0.852**

### Performance on Gene Expression and MNIST Datasets

To demonstrate the applicability of the proposed FlexGPC to other problem domains, we apply FlexGPC-MLP to the human pancreas gene expression data for cell type identification and to the MNIST dataset for the handwritten digit recognition task. The results are summarized in Table 6.

FlexGPC obtains the highest accuracy for the cell type identification task. INVASE and LSPIN achieve slightly worse accuracy of about 0.94, while gI is significantly worse, obtaining an accuracy of 0.74. We also report the two stability scores in the same table. FlexGPC gives the most stable feature selection performance. Similar conclusions can be drawn from the handwritten digit recognition task, where FlexGPC obtains the highest accuracy and stability.

**Fig. 6:**
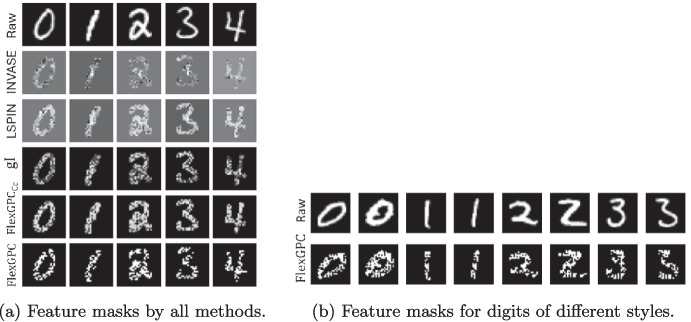
Comparison of learned feature masks for digits in MNIST

Figure 6a shows the learned feature masks based on different approaches where we observe that RAC can identify more salient regions in the image for the prediction as compared to the others. Figure 6b shows that the feature masks obtained by FlexGPC can effectively select the important pixels corresponding to the same digits written in different styles.

### Visualization of Groupings of Feature Masks

**Fig. 7:**
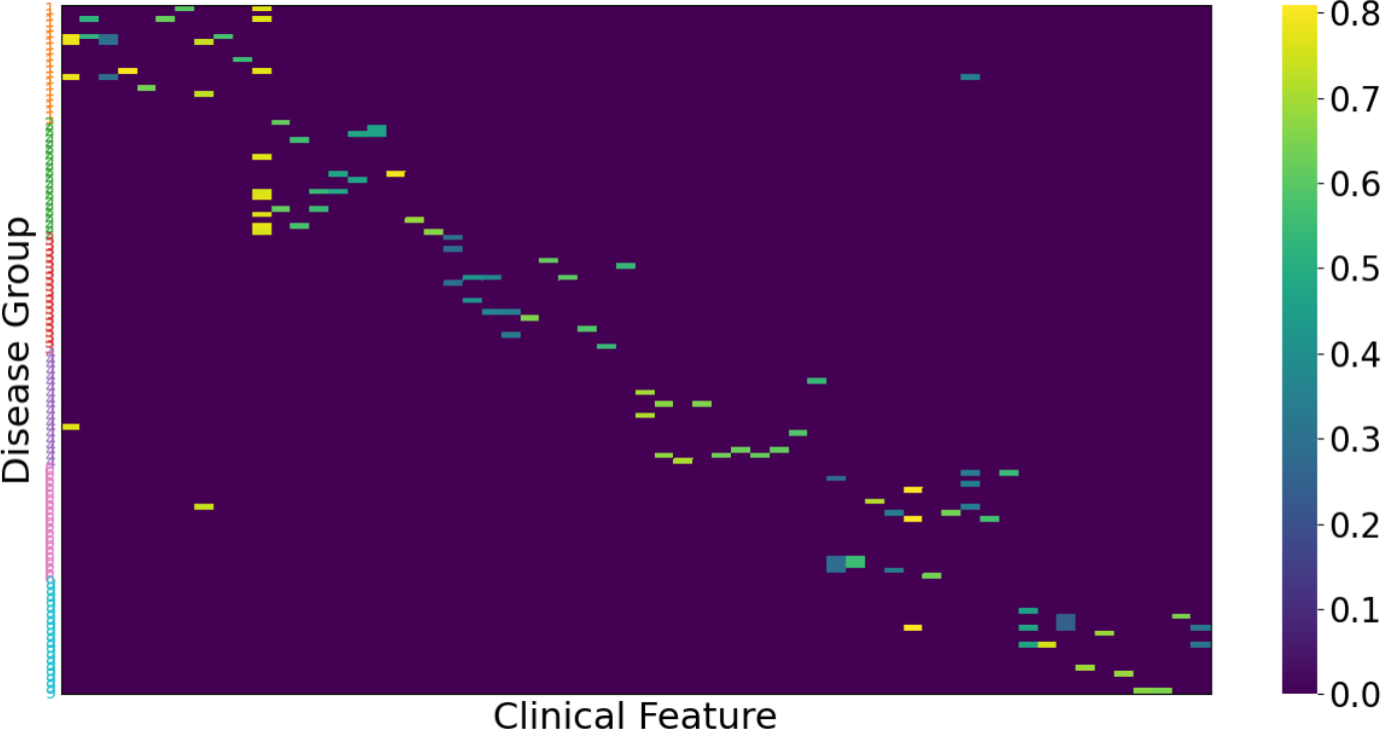
Feature masks learned from the MIMIC-III data by FlexGPC

**Fig. 8:**
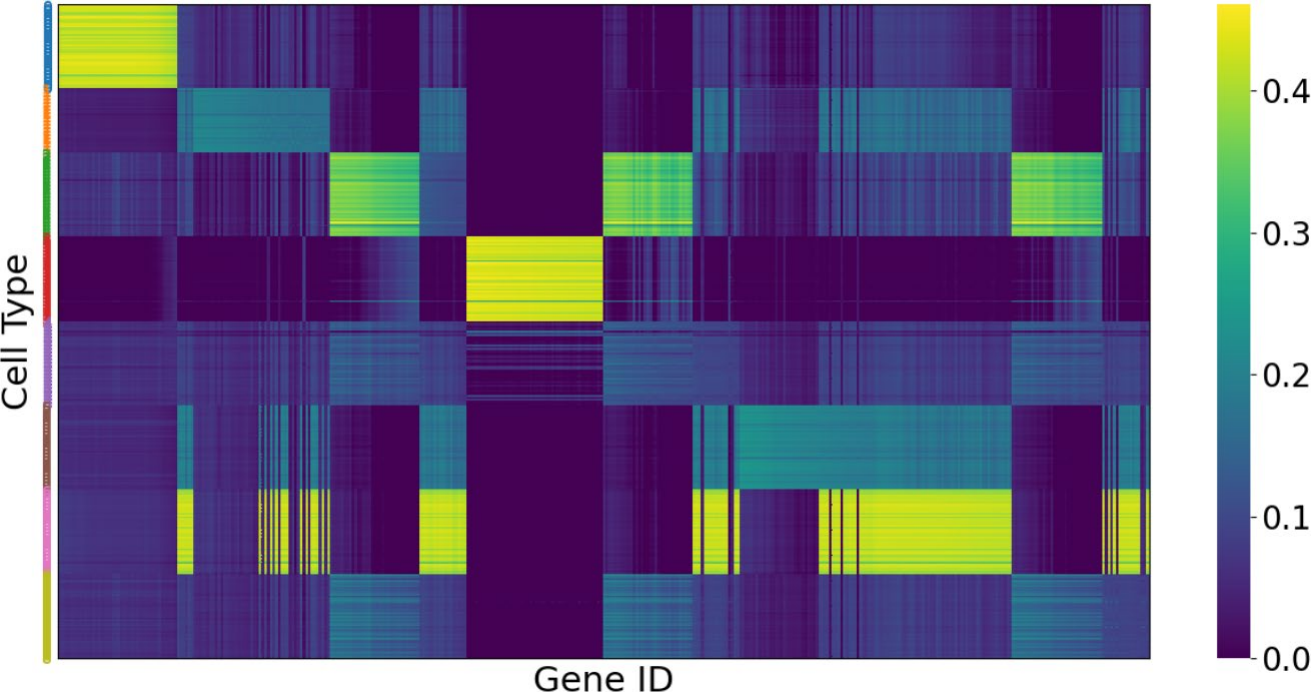
Feature masks learned from the human pancreas data by FlexGPC

Figure 7 shows the visualization of the feature masks learned by FlexGPC for the MIMIC-III dataset. To facilitate the visualization, we first apply K-means clustering to the dataset based on the hamming distance of the ground truth next-admission diagnoses to obtain the disease group label for each patient visit. The feature masks for the patient visits under the same disease group label are then presented together row-wise. In the Fig. 7, we see that the masks under the same group learned by FlexGPC share similar clinical features (histories of diagnoses and medications). For the cell type identification, we group together the cells with the same ground truth cell type label. Figure 8 shows a snapshot of feature masks learned from the gene expression data. Again, each row shows a feature mask, and the feature masks are grouped according to their ground truth cell type. We can observe that prominent and distinct gene blocks can be discovered for each cell type. This implies that FlexGPC can effectively discover the biomarkers for cells of the same type.

## Conclusion

In this paper, we propose a novel instance-wise feature grouping model called FlexGPC. We incorporated FlexGPC into different prediction models and trained them end-to-end for various analytics tasks based on electronic health records (EHR), gene expression, and image data. Across all tested tasks, FlexGPC enhanced both downstream prediction accuracy and feature selection stability. Additionally, FlexGPC offers fine-grained interpretation through the use of feature groups and increased expressiveness due to the flexible combination of feature groups. We demonstrated how FlexGPC, with a selection network implementing restricted affine combination, supports the selection and de-selection of feature groups, thereby enhancing the robustness and stability of Instance-Wise Feature Grouping (IWFG), as confirmed by extensive experiments. For future work, we plan to consider temporal information from sequential data to infer the feature mask, allowing for the exploration of dynamic interactions among input features. Additionally, developing more explicit methods to handle missing data is another direction towards achieving more robust instance-wise feature selection.

## Data Availability

The MIMIC-III data set and eICU data set can be downloaded from https://physionet.org/content/mimiciii-demo/1.4/ and https://eicu-crd.mit.edu/gettingstarted/access/ respectively. The gene expression data set is available at the following URL: https://www.ncbi.nlm.nih.gov/geo/query/acc.cgi?acc=GSM223075. And the MNIST data set is available at https://www.kaggle.com/datasets/hojjatk/mnist-dataset.

## Footnotes

1 https://www.ncbi.nlm.nih.gov/geo/query/acc.cgi?acc=GSM2230757
